# 3-(4-Chloro­phen­yl)-1-phenyl-1*H*-pyrazole-4-carbaldehyde

**DOI:** 10.1107/S1600536811023713

**Published:** 2011-06-25

**Authors:** Hoong-Kun Fun, Suhana Arshad, Shridhar Malladi, R. Selvam, Arun M. Isloor

**Affiliations:** a X-ray Crystallography Unit, School of Physics, Universiti Sains Malaysia, 11800 USM, Penang, Malaysia; b Department of Chemistry, National Institute of Technology-Karnataka, Surathkal, Mangalore 575 025, India

## Abstract

In the title compound, C_16_H_11_ClN_2_O, the chloro-substituted phenyl ring is disordered over two positions with refined site occupancies of 0.503 (2) and 0.497 (2). The dihedral angle between the pyrazole and phenyl rings is 7.93 (7)°. The pyrazole ring also forms dihedral angles of 24.43 (9)° and 28.67 (9)° with the disordered chloro-substituted benzene ring. In the crystal, mol­ecules are linked by inter­molecular C—H⋯O hydrogen bonds, generating *R*_2_^1^(7) and *R*_2_^2^(10) ring motifs. π–π inter­actions between the pyrazole and phenyl rings [centroid–centroid distance = 3.758 (1) Å] further stabilize the crystal structure.

## Related literature

For related pharmacological literature, see: Karci & Karci (2008 ▶); Isloor *et al.* (2000 ▶); Kalluraya *et al.* (2004 ▶); Isloor *et al.* (2009 ▶); Comber *et al.* (1992 ▶). For the experimental preparation, see: Vora *et al.* (2009 ▶). For reference bond-length data, see: Allen *et al.* (1987 ▶). For hydrogen-bond motifs, see: Bernstein *et al.* (1995 ▶). For stability of the temperature controller used in the data collection, see: Cosier & Glazer (1986 ▶).
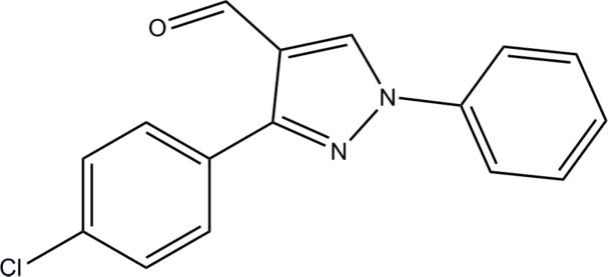


## Experimental

### 

#### Crystal data

C_16_H_11_ClN_2_O
*M* *_r_* = 282.72
Monoclinic, 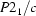

*a* = 16.0429 (4) Å
*b* = 4.8585 (1) Å
*c* = 16.7960 (4) Å
β = 96.581 (1)°
*V* = 1300.53 (5) Å^3^
*Z* = 4
Mo *K*α radiation
μ = 0.29 mm^−1^
*T* = 100 K
0.55 × 0.16 × 0.08 mm

#### Data collection

Bruker SMART APEXII CCD area-detector diffractometer
Absorption correction: multi-scan (*SADABS*; Bruker, 2009 ▶) *T*_min_ = 0.858, *T*_max_ = 0.979
26528 measured reflections
3859 independent reflections
3302 reflections with *I* > 2σ(*I*)
*R*_int_ = 0.057

#### Refinement

*R*[*F* ^2^ > 2σ(*F* ^2^)] = 0.040
*wR*(*F* ^2^) = 0.098
*S* = 1.03
3859 reflections
218 parameters
H-atom parameters constrained
Δρ_max_ = 0.36 e Å^−3^
Δρ_min_ = −0.35 e Å^−3^

### 

Data collection: *APEX2* (Bruker, 2009 ▶); cell refinement: *SAINT* (Bruker, 2009 ▶); data reduction: *SAINT*; program(s) used to solve structure: *SHELXTL* (Sheldrick, 2008 ▶); program(s) used to refine structure: *SHELXTL*; molecular graphics: *SHELXTL*; software used to prepare material for publication: *SHELXTL* and *PLATON* (Spek, 2009 ▶).

## Supplementary Material

Crystal structure: contains datablock(s) global, I. DOI: 10.1107/S1600536811023713/wn2437sup1.cif

Structure factors: contains datablock(s) I. DOI: 10.1107/S1600536811023713/wn2437Isup2.hkl

Supplementary material file. DOI: 10.1107/S1600536811023713/wn2437Isup3.cml

Additional supplementary materials:  crystallographic information; 3D view; checkCIF report

## Figures and Tables

**Table 1:** Hydrogen-bond geometry (Å, °)

*D*—H⋯*A*	*D*—H	H⋯*A*	*D*⋯*A*	*D*—H⋯*A*
C1—H1*A*⋯O1^i^	0.95	2.42	3.3545 (18)	167
C7—H7*A*⋯O1^i^	0.95	2.33	3.2684 (16)	169

Symmetry code: (i) 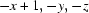.

**Fig. 1.**
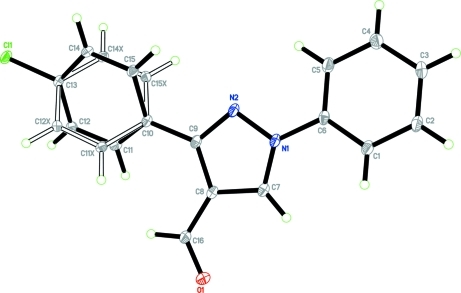
The molecular structure of the title compound, showing 30% probability displacement ellipsoids. Both disordered components are shown.

**Fig. 2.**
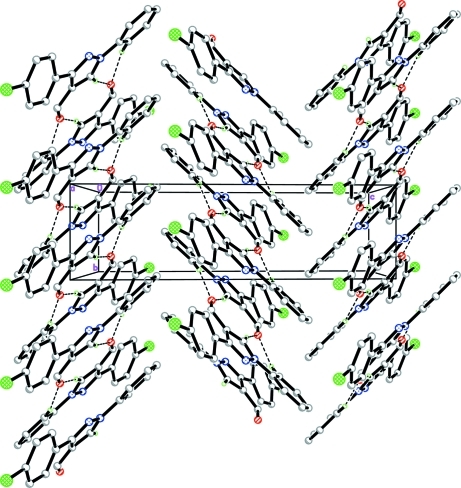
The crystal packing of the title compound. Dashed lines represent the intermolecular hydrogen bonds. Only the major disordered components are shown.

## supplementary crystallographic information

### Comment

Heterocyclic compounds have been gaining more importance in recent years due to their pharmacological activities. Nitrogen-, sulfur-, oxygen-containing five- or six-membered heterocyclic compounds are of enormous significance in the field of drug discovery process. Pyrazoles are important compounds that have many derivatives with a wide range of interesting properties, such as antipyretic, hypoglycemic, sedative-hypnotic (Karci & Karci, 2008), analgesic (Isloor *et al.*, 2000), anti-inflammatory (Kalluraya *et al.*, 2004) and antimicrobial activities (Isloor *et al.*, 2009). Much attention was paid to pyrazole as a potential antimicrobial agent after the discovery of the natural pyrazole C-glycoside and pyrazofurin which demonstrated a broad spectrum of antimicrobial activity (Comber *et al.*, 1992).

The molecular structure is shown in Fig 1. The chloro-substituted phenyl ring (C10–C15) is disordered over two positions with refined site occupancies of 0.503 (2) and 0.497 (2). The dihedral angle between the pyrazole ring (N1/N2/C7–C9) and the phenyl ring (C1–C6) is 7.93 (7)°. The pyrazole ring also forms dihedral angles of 24.43 (9)° and 28.67 (9)° with the disordered chloro-substituted phenyl rings (C10–C15) and (C10–C11X–C12X–C13–C14X–C15X), respectively. The bond lengths (Allen *et al.*, 1987) and angles are within normal ranges.

In the crystal packing (Fig. 2), the intermolecular C1—H1A···O1 and C7—H7A···O1 hydrogen bonds (Table 1) link the molecules to form dimers, generating *R*^1^_2_(7) and *R*^2^_2_(10) ring motifs (Bernstein *et al.*, 1995). π–π interactions between the pyrazole and phenyl rings further stabilize the crystal structure; [*Cg*1···*Cg*2 = 3.7579 (8) Å, where *Cg*1 and *Cg*2 are the centroids of the rings N1/N2/C7–C9 and C1–C6, respectively; symmetry code: *x*, *y* - 1, *z*].

### Experimental

Phosphoryl chloride (5 ml) was added dropwise to cold *N*,*N*- dimethylformamide (DMF) (15 ml) with continuous stirring at 273–278 K for about 30 min. 4-Chloroacetophenone phenylhydrazone (3.66 g, 15 mmol) was separately dissolved in 5 ml of DMF and was added dropwise to the former cold mixture with continuous stirring at 273–278 K for an hour. The resulting mixture was further stirred at 323–333 K for 5–6 h and cooled to room temperature. The crude product was poured into crushed ice, resulting in a white precipitate. The precipitate was filtered, washed with water and recrystallized from ethanol. Yield: 3.7 g, 87.4%. *M.p.*: 413–415 K (Vora *et al.*, 2009).

### Refinement

The chloro-substituted phenyl ring is disordered over two positions with refined site-occupancies of 0.503 (2) and 0.497 (2). All H atoms were positioned geometrically [C—H = 0.95 Å] and refined using a riding model with *U*_iso_(H) = 1.2 *U*_eq_(C).

### Crystal data

**Table:**

C_16_H_11_ClN_2_O	*F*(000) = 584
*M**_r_* = 282.72	*D*_x_ = 1.444 Mg m^−^^3^
Monoclinic, *P*2_1_/*c*	Mo *K*α radiation, λ = 0.71073 Å
Hall symbol: -P 2ybc	Cell parameters from 9960 reflections
*a* = 16.0429 (4) Å	θ = 2.4–30.2°
*b* = 4.8585 (1) Å	µ = 0.29 mm^−^^1^
*c* = 16.7960 (4) Å	*T* = 100 K
β = 96.581 (1)°	Needle, colourless
*V* = 1300.53 (5) Å^3^	0.55 × 0.16 × 0.08 mm
*Z* = 4	

### Data collection

**Table:**

Bruker SMART APEXII CCD area-detector diffractometer	3859 independent reflections
Radiation source: fine-focus sealed tube	3302 reflections with *I* > 2σ(*I*)
graphite	*R*_int_ = 0.057
φ and ω scans	θ_max_ = 30.2°, θ_min_ = 2.4°
Absorption correction: multi-scan (*SADABS*; Bruker, 2009)	*h* = −22→22
*T*_min_ = 0.858, *T*_max_ = 0.979	*k* = −6→6
26528 measured reflections	*l* = −20→23

### Refinement

**Table:**

Refinement on *F*^2^	Primary atom site location: structure-invariant direct methods
Least-squares matrix: full	Secondary atom site location: difference Fourier map
*R*[*F*^2^ > 2σ(*F*^2^)] = 0.040	Hydrogen site location: inferred from neighbouring sites
*wR*(*F*^2^) = 0.098	H-atom parameters constrained
*S* = 1.03	*w* = 1/[σ^2^(*F*_o_^2^) + (0.0397*P*)^2^ + 0.6665*P*] where *P* = (*F*_o_^2^ + 2*F*_c_^2^)/3
3859 reflections	(Δ/σ)_max_ < 0.001
218 parameters	Δρ_max_ = 0.36 e Å^−^^3^
0 restraints	Δρ_min_ = −0.35 e Å^−^^3^

### Special details

**Table:**

Experimental. The crystal was placed in the cold stream of an Oxford Cryosystems Cobra open-flow nitrogen cryostat (Cosier & Glazer, 1986) operating at 100.0 (1) K.
Geometry. All e.s.d.'s (except the e.s.d. in the dihedral angle between two l.s. planes) are estimated using the full covariance matrix. The cell e.s.d.'s are taken into account individually in the estimation of e.s.d.'s in distances, angles and torsion angles; correlations between e.s.d.'s in cell parameters are only used when they are defined by crystal symmetry. An approximate (isotropic) treatment of cell e.s.d.'s is used for estimating e.s.d.'s involving l.s. planes.
Refinement. Refinement of *F*^2^ against ALL reflections. The weighted *R*-factor *wR* and goodness of fit *S* are based on *F*^2^, conventional *R*-factors *R* are based on *F*, with *F* set to zero for negative *F*^2^. The threshold expression of *F*^2^ > σ(*F*^2^) is used only for calculating *R*-factors(gt) *etc*. and is not relevant to the choice of reflections for refinement. *R*-factors based on *F*^2^ are statistically about twice as large as those based on *F*, and *R*- factors based on ALL data will be even larger.

### Fractional atomic coordinates and isotropic or equivalent isotropic displacement parameters (Å^2^)

**Table:**

	*x*	*y*	*z*	*U*_iso_*/*U*_eq_	Occ. (<1)
Cl1	−0.055017 (18)	−0.04423 (7)	0.181350 (19)	0.02215 (9)	
O1	0.43582 (6)	−0.2143 (2)	0.07840 (6)	0.0270 (2)	
N1	0.32027 (6)	0.4298 (2)	−0.06319 (6)	0.0180 (2)	
N2	0.24330 (7)	0.4223 (2)	−0.03471 (7)	0.0190 (2)	
C1	0.41332 (9)	0.6604 (3)	−0.14732 (9)	0.0241 (3)	
H1A	0.4596	0.5576	−0.1225	0.029*	
C2	0.42428 (9)	0.8515 (3)	−0.20713 (9)	0.0248 (3)	
H2A	0.4785	0.8781	−0.2234	0.030*	
C3	0.35726 (9)	1.0028 (3)	−0.24314 (8)	0.0238 (3)	
H3A	0.3654	1.1322	−0.2840	0.029*	
C4	0.27791 (9)	0.9645 (3)	−0.21919 (8)	0.0276 (3)	
H4A	0.2318	1.0693	−0.2434	0.033*	
C5	0.26568 (8)	0.7734 (3)	−0.15994 (8)	0.0245 (3)	
H5A	0.2114	0.7462	−0.1439	0.029*	
C6	0.33339 (8)	0.6232 (3)	−0.12461 (7)	0.0183 (2)	
C7	0.37382 (8)	0.2434 (3)	−0.02733 (7)	0.0181 (2)	
H7A	0.4300	0.2126	−0.0379	0.022*	
C8	0.33170 (7)	0.1040 (3)	0.02808 (7)	0.0171 (2)	
C9	0.24994 (7)	0.2247 (3)	0.02049 (7)	0.0167 (2)	
C10	0.17600 (7)	0.1594 (3)	0.06219 (7)	0.0160 (2)	
C13	0.03440 (7)	0.0390 (3)	0.13674 (7)	0.0158 (2)	
C11	0.16431 (15)	−0.0971 (5)	0.09304 (15)	0.0177 (5)	0.503 (2)
H11A	0.2047	−0.2365	0.0871	0.021*	0.503 (2)
C12	0.09495 (15)	−0.1604 (5)	0.13298 (15)	0.0176 (5)	0.503 (2)
H12A	0.0897	−0.3359	0.1569	0.021*	0.503 (2)
C14	0.04106 (15)	0.2993 (5)	0.10535 (15)	0.0187 (5)	0.503 (2)
H14A	−0.0012	0.4336	0.1098	0.022*	0.503 (2)
C15	0.11108 (15)	0.3605 (5)	0.06696 (15)	0.0181 (5)	0.503 (2)
H15A	0.1161	0.5370	0.0436	0.022*	0.503 (2)
C11X	0.18437 (14)	0.0503 (5)	0.14160 (14)	0.0155 (5)	0.497 (2)
H11B	0.2384	0.0184	0.1695	0.019*	0.497 (2)
C12X	0.11256 (15)	−0.0091 (5)	0.17791 (14)	0.0159 (5)	0.497 (2)
H12B	0.1174	−0.0822	0.2307	0.019*	0.497 (2)
C14X	0.02656 (14)	0.1474 (5)	0.05831 (14)	0.0169 (5)	0.497 (2)
H14B	−0.0273	0.1799	0.0301	0.020*	0.497 (2)
C15X	0.09871 (15)	0.2059 (5)	0.02300 (14)	0.0173 (5)	0.497 (2)
H15B	0.0937	0.2799	−0.0297	0.021*	0.497 (2)
C16	0.36592 (8)	−0.1175 (4)	0.07881 (8)	0.0274 (3)	
H16A	0.3312	−0.1937	0.1154	0.033*	

### Atomic displacement parameters (Å^2^)

**Table:**

	*U*^11^	*U*^22^	*U*^33^	*U*^12^	*U*^13^	*U*^23^
Cl1	0.01577 (14)	0.02598 (17)	0.02634 (17)	−0.00049 (11)	0.00943 (11)	0.00063 (13)
O1	0.0174 (4)	0.0356 (6)	0.0282 (5)	0.0042 (4)	0.0033 (4)	0.0034 (4)
N1	0.0180 (5)	0.0166 (5)	0.0209 (5)	−0.0017 (4)	0.0091 (4)	−0.0018 (4)
N2	0.0182 (5)	0.0174 (5)	0.0232 (5)	−0.0007 (4)	0.0105 (4)	−0.0014 (4)
C1	0.0240 (6)	0.0170 (6)	0.0336 (7)	0.0012 (5)	0.0140 (5)	0.0014 (5)
C2	0.0267 (6)	0.0191 (6)	0.0316 (7)	−0.0023 (5)	0.0161 (5)	−0.0004 (6)
C3	0.0300 (7)	0.0229 (7)	0.0197 (6)	−0.0059 (6)	0.0082 (5)	0.0001 (5)
C4	0.0233 (6)	0.0360 (8)	0.0232 (6)	−0.0034 (6)	0.0016 (5)	0.0054 (6)
C5	0.0194 (6)	0.0331 (8)	0.0215 (6)	−0.0046 (5)	0.0041 (5)	0.0029 (6)
C6	0.0223 (6)	0.0149 (6)	0.0193 (6)	−0.0035 (5)	0.0089 (4)	−0.0032 (5)
C7	0.0170 (5)	0.0178 (6)	0.0205 (6)	−0.0017 (5)	0.0061 (4)	−0.0035 (5)
C8	0.0149 (5)	0.0190 (6)	0.0182 (5)	−0.0022 (4)	0.0048 (4)	−0.0039 (5)
C9	0.0164 (5)	0.0165 (6)	0.0180 (5)	−0.0024 (4)	0.0054 (4)	−0.0041 (5)
C10	0.0155 (5)	0.0162 (6)	0.0171 (5)	−0.0009 (4)	0.0053 (4)	−0.0024 (4)
C13	0.0136 (5)	0.0179 (6)	0.0166 (5)	−0.0007 (4)	0.0046 (4)	−0.0015 (4)
C11	0.0130 (10)	0.0192 (12)	0.0209 (12)	0.0028 (9)	0.0021 (8)	0.0000 (9)
C12	0.0169 (10)	0.0169 (12)	0.0192 (11)	−0.0001 (9)	0.0030 (8)	0.0023 (9)
C14	0.0164 (10)	0.0198 (12)	0.0205 (12)	0.0042 (9)	0.0049 (9)	−0.0010 (10)
C15	0.0189 (11)	0.0156 (11)	0.0207 (11)	0.0008 (9)	0.0059 (9)	−0.0002 (10)
C11X	0.0127 (10)	0.0170 (11)	0.0166 (11)	0.0012 (9)	0.0013 (8)	−0.0008 (9)
C12X	0.0161 (10)	0.0176 (12)	0.0145 (10)	0.0003 (9)	0.0037 (8)	0.0018 (9)
C14X	0.0127 (10)	0.0201 (12)	0.0176 (11)	0.0003 (9)	0.0005 (8)	−0.0011 (10)
C15X	0.0170 (11)	0.0197 (12)	0.0152 (11)	−0.0001 (9)	0.0020 (8)	0.0017 (9)
C16	0.0160 (6)	0.0436 (9)	0.0232 (6)	0.0018 (6)	0.0047 (5)	0.0092 (6)

### Geometric parameters (Å, °)

**Table:**

Cl1—C13	1.7405 (12)	C10—C15X	1.355 (3)
O1—C16	1.2168 (16)	C10—C11	1.371 (3)
N1—C7	1.3427 (17)	C10—C11X	1.427 (3)
N1—N2	1.3749 (13)	C10—C15	1.437 (3)
N1—C6	1.4287 (16)	C13—C14	1.379 (3)
N2—C9	1.3301 (16)	C13—C12	1.379 (3)
C1—C6	1.3909 (17)	C13—C12X	1.381 (3)
C1—C2	1.3935 (19)	C13—C14X	1.411 (3)
C1—H1A	0.9500	C11—C12	1.398 (3)
C2—C3	1.383 (2)	C11—H11A	0.9500
C2—H2A	0.9500	C12—H12A	0.9500
C3—C4	1.3908 (18)	C14—C15	1.390 (3)
C3—H3A	0.9500	C14—H14A	0.9500
C4—C5	1.391 (2)	C15—H15A	0.9500
C4—H4A	0.9500	C11X—C12X	1.394 (3)
C5—C6	1.3844 (19)	C11X—H11B	0.9500
C5—H5A	0.9500	C12X—H12B	0.9500
C7—C8	1.3877 (17)	C14X—C15X	1.389 (3)
C7—H7A	0.9500	C14X—H14B	0.9500
C8—C9	1.4292 (16)	C15X—H15B	0.9500
C8—C16	1.441 (2)	C16—H16A	0.9500
C9—C10	1.4795 (16)		
			
C7—N1—N2	112.33 (10)	C15—C10—C9	120.41 (14)
C7—N1—C6	128.90 (10)	C14—C13—C12	122.76 (16)
N2—N1—C6	118.77 (10)	C14—C13—C12X	103.95 (17)
C9—N2—N1	104.97 (10)	C12—C13—C12X	45.55 (15)
C6—C1—C2	118.74 (13)	C14—C13—C14X	45.84 (15)
C6—C1—H1A	120.6	C12—C13—C14X	101.97 (16)
C2—C1—H1A	120.6	C12X—C13—C14X	120.63 (16)
C3—C2—C1	120.88 (12)	C14—C13—Cl1	118.80 (13)
C3—C2—H2A	119.6	C12—C13—Cl1	118.43 (13)
C1—C2—H2A	119.6	C12X—C13—Cl1	119.40 (13)
C2—C3—C4	119.58 (13)	C14X—C13—Cl1	119.94 (12)
C2—C3—H3A	120.2	C10—C11—C12	122.2 (2)
C4—C3—H3A	120.2	C10—C11—H11A	118.9
C3—C4—C5	120.33 (13)	C12—C11—H11A	118.9
C3—C4—H4A	119.8	C13—C12—C11	118.1 (2)
C5—C4—H4A	119.8	C13—C12—H12A	120.9
C6—C5—C4	119.36 (12)	C11—C12—H12A	120.9
C6—C5—H5A	120.3	C13—C14—C15	118.4 (2)
C4—C5—H5A	120.3	C13—C14—H14A	120.8
C5—C6—C1	121.10 (12)	C15—C14—H14A	120.8
C5—C6—N1	118.87 (11)	C14—C15—C10	120.7 (2)
C1—C6—N1	120.02 (12)	C14—C15—H15A	119.7
N1—C7—C8	107.12 (11)	C10—C15—H15A	119.7
N1—C7—H7A	126.4	C12X—C11X—C10	119.5 (2)
C8—C7—H7A	126.4	C12X—C11X—H11B	120.3
C7—C8—C9	104.61 (11)	C10—C11X—H11B	120.3
C7—C8—C16	125.38 (11)	C13—C12X—C11X	119.6 (2)
C9—C8—C16	129.99 (11)	C13—C12X—H12B	120.2
N2—C9—C8	110.96 (10)	C11X—C12X—H12B	120.2
N2—C9—C10	118.70 (11)	C15X—C14X—C13	119.0 (2)
C8—C9—C10	130.32 (11)	C15X—C14X—H14B	120.5
C15X—C10—C11	100.24 (17)	C13—C14X—H14B	120.5
C15X—C10—C11X	119.92 (16)	C10—C15X—C14X	121.3 (2)
C11—C10—C11X	46.48 (15)	C10—C15X—H15B	119.3
C11—C10—C15	117.75 (16)	C14X—C15X—H15B	119.3
C11X—C10—C15	101.05 (15)	O1—C16—C8	125.03 (13)
C15X—C10—C9	118.24 (14)	O1—C16—H16A	117.5
C11—C10—C9	121.72 (14)	C8—C16—H16A	117.5
C11X—C10—C9	121.83 (13)		
			
C7—N1—N2—C9	0.05 (14)	C9—C10—C11—C12	179.58 (18)
C6—N1—N2—C9	179.65 (11)	C14—C13—C12—C11	−2.3 (3)
C6—C1—C2—C3	0.3 (2)	C12X—C13—C12—C11	−79.1 (3)
C1—C2—C3—C4	0.2 (2)	C14X—C13—C12—C11	42.4 (3)
C2—C3—C4—C5	−0.6 (2)	Cl1—C13—C12—C11	176.41 (17)
C3—C4—C5—C6	0.5 (2)	C10—C11—C12—C13	4.0 (3)
C4—C5—C6—C1	0.1 (2)	C12—C13—C14—C15	1.3 (3)
C4—C5—C6—N1	179.29 (13)	C12X—C13—C14—C15	47.0 (3)
C2—C1—C6—C5	−0.5 (2)	C14X—C13—C14—C15	−72.4 (2)
C2—C1—C6—N1	−179.69 (12)	Cl1—C13—C14—C15	−177.43 (17)
C7—N1—C6—C5	172.00 (13)	C13—C14—C15—C10	−1.7 (3)
N2—N1—C6—C5	−7.51 (17)	C15X—C10—C15—C14	78.7 (3)
C7—N1—C6—C1	−8.8 (2)	C11—C10—C15—C14	3.2 (3)
N2—N1—C6—C1	171.72 (12)	C11X—C10—C15—C14	−43.1 (3)
N2—N1—C7—C8	−0.27 (14)	C9—C10—C15—C14	179.34 (18)
C6—N1—C7—C8	−179.81 (12)	C15X—C10—C11X—C12X	0.4 (3)
N1—C7—C8—C9	0.36 (13)	C11—C10—C11X—C12X	−74.3 (3)
N1—C7—C8—C16	178.86 (13)	C15—C10—C11X—C12X	43.6 (3)
N1—N2—C9—C8	0.19 (13)	C9—C10—C11X—C12X	−179.63 (18)
N1—N2—C9—C10	−178.74 (10)	C14—C13—C12X—C11X	−46.5 (3)
C7—C8—C9—N2	−0.34 (14)	C12—C13—C12X—C11X	76.0 (3)
C16—C8—C9—N2	−178.75 (14)	C14X—C13—C12X—C11X	0.1 (3)
C7—C8—C9—C10	178.42 (12)	Cl1—C13—C12X—C11X	178.26 (17)
C16—C8—C9—C10	0.0 (2)	C10—C11X—C12X—C13	−0.2 (3)
N2—C9—C10—C15X	28.1 (2)	C14—C13—C14X—C15X	79.2 (3)
C8—C9—C10—C15X	−150.62 (17)	C12—C13—C14X—C15X	−45.2 (3)
N2—C9—C10—C11	152.77 (16)	C12X—C13—C14X—C15X	−0.2 (3)
C8—C9—C10—C11	−25.9 (2)	Cl1—C13—C14X—C15X	−178.35 (18)
N2—C9—C10—C11X	−151.92 (16)	C11—C10—C15X—C14X	44.8 (3)
C8—C9—C10—C11X	29.4 (2)	C11X—C10—C15X—C14X	−0.5 (3)
N2—C9—C10—C15	−23.2 (2)	C15—C10—C15X—C14X	−74.7 (3)
C8—C9—C10—C15	158.14 (16)	C9—C10—C15X—C14X	179.5 (2)
C15X—C10—C11—C12	−47.8 (3)	C13—C14X—C15X—C10	0.4 (4)
C11X—C10—C11—C12	74.0 (3)	C7—C8—C16—O1	−1.5 (2)
C15—C10—C11—C12	−4.4 (3)	C9—C8—C16—O1	176.61 (14)

### Hydrogen-bond geometry (Å, °)

**Table:**

*D*—H···*A*	*D*—H	H···*A*	*D*···*A*	*D*—H···*A*
C1—H1A···O1^i^	0.95	2.42	3.3545 (18)	167
C7—H7A···O1^i^	0.95	2.33	3.2684 (16)	169

Symmetry codes: (i) −*x*+1, −*y*, −*z*.
